# The Safety and Long-Term Efficacy of Carotid Artery Stenting: An All-Comers Registry

**DOI:** 10.7759/cureus.32060

**Published:** 2022-11-30

**Authors:** Ratheesh Kumar, Davinder Chadha, Amitoj Chaddha, Rajeev Chauhan, Navreet Singh, Pathak Kamal, Atul Mishra, Navjyot Kaur

**Affiliations:** 1 Cardiology, Army Hospital Research and Referral, New Delhi, IND; 2 Cardiology, Manipal Hospital, Bengaluru, IND; 3 Emergency, St. John’s Medical College, Bengaluru, IND; 4 Cardiology, Command Hospital Air Force, Bengaluru, IND; 5 Cardiology, Army Institute of Cardio-Thoracic Sciences, Pune, IND; 6 Radiology, Army Institute of Cardio-Thoracic Sciences, Pune, IND; 7 Radiology, Army Hospital Research and Referral, New Delhi, IND

**Keywords:** major adverse cardiac and cerebrovascular events (macces), real-world registry, stroke, long term, carotid artery stenting

## Abstract

Background

Carotid artery stenting (CAS) has emerged as a less invasive alternative to carotid endarterectomy (CEA) for the prevention of future cerebrovascular events in patients with carotid artery stenosis. Despite multiple randomized controlled trials (RCTs) comparing CAS and CEA for carotid disease, real-world data outside the rigorous environment of trials is scarce.

Methods

The present study is a prospective observational study conducted at a tertiary care center, wherein all patients who underwent CAS between January 2007 and December 2019 were included. All patients were followed up for one year of the last enrolled patient at an interval of one, six, and 12 months and then yearly thereafter. The primary composite outcome was defined as a combination of periprocedural (until 30 days of procedure) major adverse cardiac and cerebrovascular events (MACCEs) and the long-term incidence of ipsilateral stroke. The secondary outcome included the rate of restenosis.

Results

A total of 115 patients (86 males and 29 females) (147 lesions) who underwent CAS between 2007 and 2019 were followed up for a median of 80.5 months. Seventy-seven (67.27%) patients were symptomatic, and 38/115 (33%) were asymptomatic. Periprocedural MACCEs were noted in six patients, and four patients had ipsilateral stroke on long-term follow-up; hence, the primary composite outcome was observed in 10 (8.7%) patients. Higher age was found to be significantly associated with the primary composite outcome (p-value = 0.005). Five (4.34%) patients were lost to follow-up, while four (3.48%) patients developed restenosis.

Conclusion

CAS is a safe and less invasive intervention in patients with significant carotid artery stenosis and is equally effective in preventing future strokes. The incidence of primary outcome rises with an increase in age.

## Introduction

Carotid artery stenosis contributes to around 20%-30% of all ischemic strokes [1]. In the last two decades, carotid artery stenting (CAS) has emerged as a less invasive alternative to carotid endarterectomy (CEA) for the prevention of future cerebrovascular events in patients with carotid artery stenosis [2]. The data on the effectiveness of CAS when tested against the gold standard CEA remains far from being conclusive. Few trials have shown that when CAS is done using an embolic protection device (EPD), it is non-inferior to CEA [1,3,4]. There are trials such as the International Carotid Stenting Study (ICSS) [5] and the Endarterectomy Versus Angioplasty in Patients With Symptomatic Severe Carotid Stenosis (EVA-3S) [6] that showed that the risk of stroke and periprocedural death is higher with CAS than with CEA. All these trials were heterogenous in patient selection criteria and included both symptomatic and asymptomatic individuals [7]. Besides, the skill and expertise of operators differed, and the use of EPD and dual antiplatelets were inconsistent. A recent meta-analysis [8] involving 9,753 participants found that CAS in symptomatic carotid artery stenosis was associated with a higher risk of death and stroke within 30 days of the procedure, whereas CEA increased the risk of myocardial infarction (MI), cranial nerve palsy, and access site hematoma. However, a prespecified subgroup analysis showed that the risk of periprocedural death and stroke was not higher in patients less than 70 years with CAS [8]. The two modalities were comparable in patients with asymptomatic carotid artery stenosis.

Carotid artery stenosis is a disease of the elderly, and such patients usually have multiple comorbidities. CAS is definitely a lesser invasive alternative to CEA for carotid artery stenosis. Over the years, the expertise of interventionists has improved, the hardware has evolved, and EPDs have been introduced. Besides, most of the data on CAS is quoted from randomized controlled trials (RCTs) with stringent inclusion and exclusion criteria, which may not be applicable to our daily clinical practice. The data on CAS from the Indian subcontinent is even more sparse [9,10]. This study was done to evaluate the safety and efficacy of CAS in patients with carotid artery stenosis in a real-world scenario.

## Materials and methods

Study design

This was a prospective observational study wherein all patients with carotid artery stenosis who underwent CAS at a tertiary care center between January 2007 and December 2019 were included after obtaining informed consent of patients or next of kin. Ethical clearance for the publication of data was taken from the Institutional Ethics Committee of Command Hospital Air Force (CHAF-IEC 46/07) in February 2007. The inclusion criteria for CAS included all neurologically symptomatic patients (transient ischemic attack (TIA), non-disabling stroke, or amaurosis fugax in the last six months) with >70% stenosis of the corresponding carotid artery by Doppler ultrasound (DUS) or computed tomography angiography (CTA) or magnetic resonance angiography (MRA) or >50% stenosis by angiogram [11]. Asymptomatic patients were included if they were incidentally detected to have >60% stenosis by angiogram, >70% stenosis by DUS, or > 80% stenosis by CTA or MRA [11]. The exclusion criteria included unfavorable anatomy precluding intervention, bleeding disorders, pregnancy, stage III chronic kidney disease with estimated glomerular filtration rate (eGFR) < 60 mL/minute/1.73 m^2^, and complex lesions such as chronic total occlusion (CTO) or heavily calcified or thrombus-laden lesions. The demographic characteristics of all included patients were noted along with atherosclerotic risk factors. The initial neurological examination and that before hospital discharge were noted for all included patients using the National Institute of Health Stroke Scale (NIHSS) [12].

Procedure protocol

All CAS were performed by the interventionists who had adequate experience in performing the procedure with at least 25 independent cases. All included patients received aspirin 150 mg/day, clopidogrel 75 mg/day, and statin starting at least one week before the procedure. Unfractionated heparin (UFH) was given intravenously before the procedure and titrated to keep the activated clotting time between 200 and 250 seconds. After assessing the lesion characteristics, EPD was placed in the distal-most cervical or petrous segment of the internal carotid artery. In the case of tight lesions, it was pre-dilated with a 3-mm angioplasty balloon before placing a filter. The lesions were stented using different types of self-expandable stents as per operator preference and lesion characteristics. These included open-cell design, closed-cell design, and hybrid stents. Nitrate infusion, atropine, mephentermine, and a temporary pacemaker were kept readily available. Technical success was defined as restoration of cerebral flow through the lesion, with >20% improvement in stenosis and residual stenosis of <50%. Stroke was defined as major (i.e., a new focal ischemic neurological deficit (FND) of abrupt onset, which lasts for more than 24 hours and increases the patient’s NIHSS score by at least 4) or minor (i.e., a new FND of abrupt onset, lasting at least 24 hours and not meeting the major stroke definition). MI was defined as the elevation of creatine kinase-MB (CK-MB)/troponin > 2 upper reference limits of normal in addition to chest pain or symptoms consistent with ischemia or electrocardiogram (ECG) changes. Minor adverse events were self-explanatory and were defined as all those that did not require any intervention.

Follow-up

All included patients were followed up for one year of the last enrolled patient at an interval of one, six, and 12 months and then yearly thereafter. The TIA questionnaire [13] and NIHSS assessment score [12] were used to evaluate any new FND. The primary composite outcome was defined as a combination of periprocedural major adverse cardiac and cerebrovascular events (MACCEs) and the long-term incidence of ipsilateral carotid artery stroke. Periprocedural MACCE included any stroke corresponding to carotid artery intervention, MI, or death within 30 days of the procedure. The rate of restenosis was considered the secondary outcome. All patients were subjected to DUS examination at an interval of 12 months irrespective of symptomatic status. If there was any alteration in DUS (peak systolic velocity > 3 m/second), a selective carotid angiogram was performed. Restenosis was defined as intrastent stenosis > 50% by carotid angiogram. All patients were treated with optimal medical therapy. Clopidogrel was stopped after 30 days of the procedure, while aspirin was continued indefinitely. All atherosclerotic risk factors were controlled as per the existing guidelines.

Statistical methods

Data analysis was done using the R software version 4.1.0. Quantitative data were expressed using means, medians, and standard deviations (SD), and qualitative data were expressed using frequencies and percentages. The association between co-variables and MACCEs/primary composite outcome was determined using logistic regression analysis. All tests were considered statistically significant if the p-value was less than 0.05. Freedom from the primary composite outcome at the end of the study period was also presented using the Kaplan-Meier plot.

## Results

A total of 115 patients who underwent CAS (86 (74.78%) males and 29 (25.22%) females) were included in the study between 2007 and 2019. The total number of lesions that were intervened was 147. The mean age of the enrolled patients was 62.15 (±6.97) years. A total of 77/115 (66.95%) patients were symptomatic, and 38/115 (33.04%) were asymptomatic. Ninety-four (81.7%) patients had hypertension, and 52 (45.2%) patients were diabetic. Thirty (26.08%) patients had non-carotid peripheral artery disease (PAD), and 78 (67.82%) patients had coronary artery disease. The majority of the lesions (132/147 (93.83%)) had >70% stenosis. Twenty-six (22.60%) patients had bilateral carotid artery lesions. EPDs were used in all patients, with a single filter in 102 (88.7%) patients and two filters in 13 (11.3%) patients. Two filters in a single patient were used when bilateral carotid arteries were addressed. A total of 134 stents (76 open-cell design stents, nine closed-cell design stents, and 49 hybrid design stents) were used. One stent each was used in 97 (84.34%) patients, two stents in 14 (12.17%) patients, and three stents in three (2.6%) patients. In those with bilateral carotid artery stenosis, the interventions were spaced out by at least one-week intervals.

Procedural success was obtained in 114 (99.13%) patients. In one patient, stenting could not be done due to excessive proximal tortuosity. Table 1 summarizes the cohort’s demographics and procedural characteristics.

**Table 1:** Demographics and procedural characteristics of the study cohort SD, standard deviation; CAD, coronary artery disease; PCI, percutaneous coronary intervention; CABG, coronary artery bypass graft; TVD, triple-vessel disease; LMCA, left main coronary artery disease; PAD, peripheral artery disease; TIA, transient ischemic attack

Characteristic (n = 115)	Number (%)
Mean age in years (±SD)	62.15 ± 6.97
Male	86 (74.8)
Hypertension	94 (81.7)
Diabetes mellitus	52 (45.2)
Smoking	27 (23.5)
Dyslipidemia	19 (16.5)
Family history of CAD	6 (5.2)
History of CAD	
Post-PCI	26 (22.6)
Post-CABG	15 (13.1)
TVD	26 (22.6)
LMCA	11 (9.6)
Indication for carotid stenting (n = 115)	
Symptomatic	77 (66.9)
Hemiparesis	48 (41.7)
TIA	29 (25.2)
Asymptomatic	38 (33)
Carotid lesion severity (n = 147)	
<70%	15 (10.27)
≥70%	132 (93.83)
Site of involvement (n = 115)	
Unilateral	89 (77.39)
Bilateral	26 (22.60)
Lesion characteristics (n = 115)	
Calcification	2 (1.7)
Ulceration	5 (4.3)
Tortuosity	1 (0.9)
Thrombus	1 (0.9)
Type of stent used (n = 134)	
Open-cell design	76 (56.71)
Closed-cell design	9 (6.71)
Hybrid design	49 (36.56)
Post-dilatation (n = 115)	
No	29 (25.2)
Yes	86 (74.8)

MACCEs, defined by ipsilateral stroke, MI, or death at the end of 30 days, were noted in six (5.22%) patients. Minor ipsilateral ischemic stroke was noted in three (2.61%) patients, and major ipsilateral ischemic stroke was seen in two (1.73%) patients. One patient with hypertension and symptomatic right internal carotid artery stenosis (95%) with left hemiparesis developed hyper-perfusion syndrome and right basal ganglia hemorrhagic stroke post-CAS. One symptomatic patient with diabetes mellitus, hypertension, and bilateral carotid stenosis who was subjected to bilateral CAS had a procedural complication in the form of stuck EPD (Angioguard^TM^, Cordis Endovascular, FL, USA) on the right side. The EPD was left in situ with a full dose of anticoagulation. Other minor complications such as TIA, bradycardia, hypotension, carotid arterial spasm, and puncture site hematoma were noted in a few patients as mentioned. Table 2 summarizes the short- and long-term clinical outcomes of the study cohort. On logistic regression analysis, age < 70 years (p-value = 0.0013, odds ratio = 0.026, confidence interval (CI) = 0.003-0.240) and post-dilatation (p-value = 0.0382, odds ratio = 0.156, CI = 0.027-0.904) were significantly associated with lower MACCE. Among the various co-variables, only age < 70 years was found to be significantly associated with the lower primary composite outcome (p-value = 0.005, odds ratio = 0.081, CI = 0.020-0.334) (Table 3).

**Table 2:** Short- and long-term clinical outcomes of the study cohort MACCE, major adverse cardiac and cerebrovascular event; MI, myocardial infarction; TIA, transient ischemic attack

Event	Periprocedural (30 days) (number (%))		Long term (31 days up to 12 years) (number (%))
Procedural success	114 (99.1)		-
Minor ipsilateral stroke	3 (2.6)		3 (2.7)
Major ipsilateral stroke	2 (1.7)		1 (0.9)
Death	-		6 (5.5)
MI	1 (0.9)		1 (0.9)
TIA	2 (1.7)		-
Restenosis	-		4 (3.6)
Cerebral hyper-perfusion syndrome	1 (0.9)		-
Bradycardia/hypotension	26 (22.60)		-
Stuck filter	1 (0.9)		-
Puncture site hematoma	3 (2.6)		-
Pseudoaneurysm (vascular access)	3 (2.6)		-
Carotid spasm	4 (3.5)		-
MACCE (either periprocedural, nonfatal stroke, MI, or death)	6 (5.2)		
Long-term ipsilateral stroke (30 days to 12 years)	-		4 (3.6)
Primary outcome (either periprocedural, nonfatal stroke, MI, or death or long-term ipsilateral stroke)		10 (8.7)	
Asymptomatic restenosis		4 (3.5)	
Lost to follow-up		5 (4.4)	

**Table 3:** Association of co-variables with MACCE and the primary composite outcome CAD, coronary artery disease; CL, confidence limits; DM, diabetes mellitus type 2; EPD, embolic protection device; LMCA, left main coronary artery; MACCE, major adverse cardiac and cerebrovascular events; PAD, peripheral artery disease; TVD, triple-vessel disease

Parameter	Effect	MACCE			Primary composite outcome		
		Odds ratio	CL (upper-lower)	p-value	Odds ratio	CL (upper-lower)	p-value
Age category (years)	<70 versus ≥70	0.026	0.003-0.240	0.0013	0.081	0.020-0.334	0.0005
Sex	Female versus male	3.200	0.607-16.866	0.1702	1.303	0.313-5.423	0.7160
DM	Yes versus no	0.571	0.100-3.256	0.5285	1.196	0.326-4.387	0.7876
Dyslipidemia	Yes versus no	1.036	0.114-9.428	0.9752	1.328	0.258-6.840	0.7341
LMCA involvement	Yes versus no	2.134	0.224-20.305	0.5097	1.328	0.258-6.840	0.7341
TVD	Yes versus no	1.783	0.307-10.354	0.5196	0.848	0.168-4.275	0.8415
CAD	Yes versus no	1.500	0.263-8.549	0.6482	1.801	0.440-7.367	0.4129
PAD	Yes versus no	0.800	0.089-7.218	0.8425			
Carotid ulceration	Yes versus no				2.696	0.272-26.753	0.3970
Carotid calcification	Yes versus no	1.709	0.183-15.991	0.6385	1.854	0.352-9.777	0.4667
Pre-dilation	Yes versus no	0.363	0.070-1.875	0.2264	-	-	-
Post-dilation	Yes versus no	0.156	0.027-0.904	0.0382	0.312	0.083-1.168	0.0838
Number of lesions	-	0.600	0.071-5.067	0.6387	1.959	0.564-6.803	0.2898
Number of vessels	-	-	-	-	1.468	0.385-5.602	0.5744
Number of stents	-	0.267	0.010-6.872	0.4252	0.797	0.150-4.222	0.7895
Number of EPDs	-	-	-	-	1.011	0.116-8.827	0.9920
Stent length	-	1.021	0.896-1.163	0.7589	1.013	0.923-1.111	0.7860
Stent diameter	-	1.438	0.637-3.247	0.3818	1.459	0.766-2.781	0.2509

**Figure 1:**
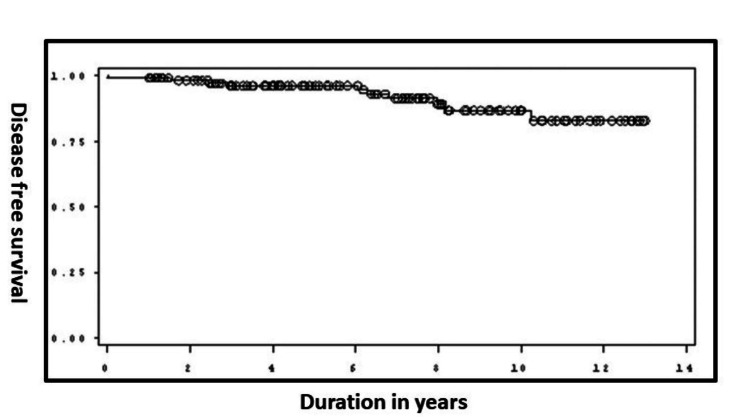
Kaplan-Meier plot for freedom from the primary composite outcome at the end of the study period

All patients were followed up for a median of 80.5 (range: 17-155) months. A total of five (4.34%) patients were lost to follow-up. An additional four (3.6%) patients had ipsilateral stroke on long-term follow-up; hence, the primary composite outcome was observed in 10 (8.7%) patients. On long-term follow-up, six deaths occurred; however, none of them were stroke-related. There were no deaths in the periprocedural period. Four (3.47%) patients developed asymptomatic restenosis on follow-up. No intervention was done in any of these patients. Figure 1 depicts the Kaplan-Meier plot that shows that around 75% of the cohort did not have primary endpoint occurrence for around 13 years from the date of the procedure. The presence of any no carotid PAD was significantly associated with restenosis (p-value = 0.0260, odds ratio = 13.896, CI = 1.370-140.945).

## Discussion

The current study presents a real-world experience of CAS in patients with carotid artery stenosis irrespective of the symptom status. The included patients were followed up for a median of 80.5 months. The mean age of our cohort (62.15 ± 6.97 years) was lower than other trials, which studied carotid artery intervention (Stent versus Surgery for Asymptomatic Carotid Stenosis (ACT 1) (67.7 ± 7 years) [14], Carotid Revascularization Endarterectomy versus Stenting Trial (CREST) (68.9 ± 9 years) [3,4], and Stenting and Angioplasty with Protection in Patients at High Risk for Endarterectomy (SAPPHIRE) study (72.5 ± 8.3 years) [15]). In our study, the incidence of diabetes was found to be around 45.2%, which was more than those reported in other trials such as ACT 1 (35.6%) [14], CREST (30.6%) [3,4], and SAPPHIRE (25.3%) [15]. However, the incidence of dyslipidemia was found to be less (16.5%) compared to other studies such as ACT 1 (90%) [14], CREST (78.9%) [3,4], and SAPPHIRE (78.5%) [15].

In the index study, the carotid stent was implanted in all patients except one, where extreme tortuosity of the internal carotid led to the failure of stent implantation. In the current study, six (5.21%) patients were noted to have MACCE at the end of 30 days. The MACCE rate of our study is comparable with that of the CREST trial (7%) [3] but higher than the ACT 1 trial (3.3%) [14], SAPPHIRE study (4.8%) [15], and CArotid Stenting Boston Scientific SurveillANce ProgrAm (CABANA) trial (4.6%) [16]. Ronchey et al. recorded a very low 30-day MACCE rate of 2.1% [17]. The incidence of periprocedural stroke and MI in our study was comparable to other studies, except for the SAPPHIRE study, which reported a higher rate of MI (2.4%) [15]. This may probably be explained by the fact that the mean age of patients in the SAPPHIRE trial was a decade more than the index study (72 years). No periprocedural deaths were reported in the present study, but a variable number of the same have been noted in other studies [3,4,14-16,18]. Hyper-perfusion syndrome leading to hemorrhagic stroke was noted in one patient in our study, which has been reported earlier [19].

Our study revealed an ipsilateral stroke rate (beyond 30 days) of 3.48% at the end of 12 (median: 6) years. This is much less than the stroke incidence seen in the CREST trial (10.8% at 10 years) [4] and SAPPHIRE trial (15.2% at five years) [15]. The primary outcome (combination of periprocedural outcome along with long-term ipsilateral stroke) was seen in 10 (9.1%) patients in our study, which was less than observed in the CREST (11.8% at 10-year follow-up) [4] and Endarterectomy Versus Angioplasty in Patients With Severe Symptomatic Carotid Stenosis (EVA-3S) (11.5% at 10-year follow-up) trial [20]. The low incidence of stroke in our study could be due to the younger age group of the patients and stringent follow-up with strict control of their comorbidities. Age was found to be significantly associated with the primary outcome in our study. Restenosis was seen in four (3.48%) patients, which was considerably lower than seen in the International Carotid Stenting Study (ICSS) randomized trial (10.8% at five years) [21] and EVA-3S trial (5% at 10 years) [20] and in the study by Mayoral et al. (4.4% at 15 years) [18]. In the present study, logistic regression analysis revealed PAD to be significantly associated with restenosis. Table 4 compares the short- and long-term outcomes of our study with other major trials. Unlike most of the other studies, we used all types of available stent cell designs (open, closed, and hybrid) and found no difference between the periprocedural events or long-term outcomes between the different stent cell designs. This is in contrast to the study done by Bosiers et al. who reported a significant decrease in periprocedural events in symptomatic patients of carotid artery stenosis with the use of a closed-cell design stent (free cell area < 2.5 mm^2^) [22].

**Table 4:** Comparative analysis of short- and long-term outcomes in the present study versus other trials and study ACT 1, Stent versus Surgery for Asymptomatic Carotid Stenosis; CABANA, Carotid Stenting Boston Scientific Surveillance Program; CREST, Carotid Revascularization Endarterectomy versus Stenting Trial; EVA-3S, Endarterectomy Versus Angioplasty in Patients With Severe Symptomatic Carotid Stenosis; MI, myocardial infarction; SAPPHIRE, Stenting and Angioplasty with Protection in Patients at High Risk for Endarterectomy; MACCE, major adverse cardiac and cerebrovascular event

	Present study (n = 115)	CREST [3,4] (n = 1,262)	ACT 1 [14] (n = 1,089)	SAPPHIRE [15] (n = 167)		EVA-3S [19] (n = 265)		CABANA [16] (n = 1,025)	Mayoral et al. [18](n = 344)	Ronchey et al. [17] (n = 877)
30-day outcome										
Stroke (%)	4.3	4.1	2.8	3.6	9.05		3.3		2.3	1.8
MI (%)	0.9	1.1	0.5	2.4	0.37		0.5		0	0.1
Death (%)	-	0.7	0.1	1.2	0.75		1.3		0.3	0.2
MACCE (periprocedural stroke, MI, death) (%)	5.2	5.2	3.3	4.8	10.18		4.6		2.3	2.1
Long-term outcome										
Follow-up (year)	6.7	10	-	1	10		-		4	3
Stroke (%)	3.48	10.8		6.2	16.9				5.5	-
MACCE, ipsilateral stroke (%)	8.7	11.8	-	12.2	12.5		-		4.6	-
Periprocedural stroke, death, ipsilateral stroke (%)	7.8	-	-	5.5	11.5		-		4.6	-
Restenosis (%)	3.48	12.2	-	-	2.6		-		4.4	3.2

EPDs were used in all patients in the current study. None but one patient had EPD-related complications. A meta-analysis of 24 studies reported that EPD use was associated with a 41% decrease in embolic stroke [23]. A prospective registry showed that the use of EPD during CAS reduces in-hospital death and stroke from 4.9% to 2.1% (p = 0.004) [24]. The results of our study are consistent with other studies that showed a reduced incidence of MACCE with EPD.

The current study was done to provide real-world data on the safety and efficacy of CAS in patients with carotid stenosis. Unlike the RCTs with strict inclusion criteria, such registries provide more realistic data that can be applied to daily clinical practice and also test the safety and feasibility of a given intervention outside the trial environment. This is one of the few registries of CAS from the Indian subcontinent with long-term follow-up. The study population comprised both symptomatic and asymptomatic patients of carotid artery stenosis who were at standard surgical risk. Besides using EPDs in all carotid interventions, all efforts were made to address the atherosclerotic risk factors of patients during follow-up. This study reproduced the low rates of MACCE and the low long-term risk of ipsilateral stroke and restenosis after CAS as shown in various RCTs.

Limitations

The major limitation of our study is the small sample size. Due to the small sample size and low event rate, the true association between various co-variables and the primary outcome might not have been accurately assessed. We did not follow up with patients who were managed medically or were sent for CEA after heart team discussion, which could have added further value to the study. The study spans over 12 years, during which there has been tremendous advancement in the hardware and expected change in procedural technique in the study population between initial patients and the more recent ones.

## Conclusions

CAS is safe and feasible in selected patients with carotid stenosis who warrant intervention as per the current guidelines irrespective of the symptom status. The incidence of primary outcome consisting of MACCE at 30 days of the procedure and long-term ipsilateral stroke rises with an increase in age.
